# Cytotoxicity of Venoms and Cytotoxins from Asiatic Cobras (*Naja kaouthia*, *Naja sumatrana*, *Naja atra*) and Neutralization by Antivenoms from Thailand, Vietnam, and Taiwan

**DOI:** 10.3390/toxins14050334

**Published:** 2022-05-10

**Authors:** Ho Phin Chong, Kae Yi Tan, Bing-Sin Liu, Wang-Chou Sung, Choo Hock Tan

**Affiliations:** 1 Venom Research and Toxicology Laboratory, Department of Pharmacology, Faculty of Medicine, University of Malaya, Kuala Lumpur 50603, Malaysia; schp_182@hotmail.com; 2 Protein and Interactomics Laboratory, Department of Molecular Medicine, Faculty of Medicine, University of Malaya, Kuala Lumpur 50603, Malaysia; kytan_kae@um.edu.my; 3 National Institute of Infectious Diseases and Vaccinology, National Health Research Institutes, Miaoli 35053, Taiwan; bingsin@nhri.edu.tw

**Keywords:** cytotoxin, Chinese Cobra, Monocled Cobra, Equatorial Spitting Cobra, in vitro assay

## Abstract

Envenoming by cobras (*Naja* spp.) often results in extensive local tissue necrosis when optimal treatment with antivenom is not available. This study investigated the cytotoxicity of venoms and purified cytotoxins from the Monocled Cobra (*Naja kaouthia*), Taiwan Cobra (*Naja atra*), and Equatorial Spitting Cobra (*Naja sumatrana*) in a mouse fibroblast cell line, followed by neutralization of the cytotoxicity by three regional antivenoms: the Thai *Naja kaouthia* monovalent antivenom (NkMAV), Vietnamese snake antivenom (SAV) and Taiwanese Neuro bivalent antivenom (NBAV). The cytotoxins of *N. atra* (NA-CTX) and *N. sumatrana* (NS-CTX) were identified as P-type cytotoxins, whereas that of *N. kaouthia* (NK-CTX) is S-type. All venoms and purified cytotoxins demonstrated varying concentration-dependent cytotoxicity in the following trend: highest for *N. atra*, followed by *N. sumatrana* and *N. kaouthia*. The antivenoms moderately neutralized the cytotoxicity of *N. kaouthia* venom but were weak against *N. atra* and *N. sumatrana* venom cytotoxicity. The neutralization potencies of the antivenoms against the cytotoxins were varied and generally low across NA-CTX, NS-CTX, and NK-CTX, possibly attributed to limited antigenicity of CTXs and/or different formulation of antivenom products. The study underscores the need for antivenom improvement and/or new therapies in treating local tissue toxicity caused by cobra envenomings.

## 1. Introduction

Snakebite envenoming is a priority neglected tropical disease according to the World Health Organization [1,2]. Each year, approximately 83,000–138,000 deaths and 3 times as many limb amputations or other permanent disabilities are caused by snakebite envenoming [1]. In Asia and Africa, cobras (*Naja* spp.) are commonly listed under Category 1 of medically important venomous snakes by the WHO as they are frequently implicated in snakebite envenoming and associated with high mortality and morbidity [3,4]. Envenoming caused by cobras (*Naja* spp.) can result in systemic neurotoxicity and local tissue necrosis at the bite site and beyond [3,5,6]. In addition, venom ophthalmia can occur if venoms are sprayed by “spitting” cobras into the victim’s eyes, resulting in ocular injuries and blindness [7,8,9]. The immense tissue-damaging effect of cobra venoms is attributed to the cytotoxins (CTX) present in virtually all cobra venoms, constituting 20–80% of total venom proteins [5,10,11,12]

Cytotoxins are non-enzymatic, highly basic (pI > 10) β-sheet single-chained polypeptides consisting of 60–70 amino acid residues and have a molecular weight of ~6.5–7.0 kDa. Structurally, they have a three-fingered loop-folding topology reinforced by four disulfide bonds between eight conserved cysteine residues [13,14]. CTXs exhibit strong amphiphilic properties on their molecular surface and the hydrophobic tips of the three-finger structure flanked by polar residues are shown to be the principal membrane-binding motif [15], in particular toward the anionic phosphatidylserine lipid [16]. Previous studies proposed 2 distinct types of CTX, i.e., the P-type and S-type CTXs, where the former is characterized by the presence of Pro-31 within a putative phospholipid binding site near the tip of loop 2, while S-type CTXs are characterized by the presence of Ser-29 within the same but more hydrophilic region [15,17]. CTXs have been previously shown to be highly cytotoxic in vitro toward various mammalian cell lines (both cancer and non-cancer) [18,19,20] and are able to induce dermonecrosis in mice [21,22]. This is consistent with the local tissue damage reported in most cobra bite envenoming and is a cause of limb deformity, amputation, and permanent disabilities when treatment is inadequate [23,24].

To date, antivenoms remain the definitive treatment for snakebite envenoming. The gold standard advocated by the WHO for antivenom efficacy assessment is the neutralization of lethality in rodents, an assay that essentially tests against the systemic toxicity of venom under acute conditions [25]. The efficacy of antivenom in neutralizing cobra CTX-induced cytotoxicity, however, has not been commonly addressed, presumably because of the general perception that antivenom is effective systemically but has limitations in the treatment of local envenoming [2,26]. Nevertheless, antivenom treatment is still recommended by most management guidelines to alleviate the effect of local envenoming, indicated when there is rapid swelling that extends beyond a joint or involves half the limb [3,5]. It is therefore important to assess the efficacy of antivenom in neutralizing the cytotoxic activity of different cobra venoms and, importantly, the causative cytotoxins contained therein. Preclinically, a mouse model is used to demonstrate dermonecrosis induced by snake venoms and to serve as a model for neutralization studies [27,28,29]. The development of dermonecrosis takes up to a few days and has been deemed rather controversial nowadays from an animal ethics point of view. Many authorities, including the WHO, encourage implementing the 3Rs Principle (3R: replacement, reduction, and refinement) in antivenom production and assessment procedures before the use of in vivo models [25,30]. Therefore, in vitro approaches such as cell-based assays may be opted as a surrogate model for venom-induced cytotoxicity [31,32,33]. With apt modifications, the in vitro assays can be applied to examine the neutralization efficacy of antivenom against CTX-induced necrosis while minimizing animal use. Therefore, the present study aimed to investigate the cytotoxic activities of the venoms and purified CTX from three Asiatic cobra species known for causing significant local tissue damage, Monocled Cobra (*Naja kaouthia*), Equatorial Spitting Cobra (*Naja sumatrana*), and Taiwanese or Chinese Cobra (*Naja atra*), through a cell-based assay using rodent fibroblast cell line (CRL-2648) derived from the subcutaneous tissue of the skin.

The present study further investigated the neutralization efficacy of the *Naja kaouthia* Monovalent Antivenom (NkMAV, Thailand and SAV, Vietnam) and the Neuro Bivalent Antivenom raised against *Naja kaouthia* and *Bungarus multicintus* (NBAV, Taiwan) against the cytotoxicity of the cobra venoms and CTX. The three antivenoms were studied as they are the regional cobra antivenoms available and clinically used for cobra envenoming. Also, they are different in terms of formulation (monovalent vs. bivalent) and the venom immunogen used in raising the antivenoms. Thai *N. kaouthia* monovalent antivenom (NkMAV) and Vietnamese Snake antivenom (as per manufacturer’s label, SAV) are monovalent antivenoms (raised against *N. kaouthia*), and Taiwanese neurobivalent antivenom (NBAV) is a bivalent antivenom raised against *N. atra* and *Bungarus multicinctus*). The difference in the formulation might affect the specific antibody concentration and, thus, the efficacy against specific CTX of different species. In terms of CTX, *N. kaouthia* venom contains mainly S-type, while *N. atra* and *N. sumatrana* have mainly P-type CTX [32,34,35]. Therefore, the antivenoms produced from different venoms could have varied efficacy against the two CTX subtypes from different species. Hence, the comparison of the efficacy of the three antivenoms against the different CTX subtypes may provide insights into the optimization of antivenom use for the treatment of local necrosis caused by cobra envenoming.

## 2. Results

### 2.1. Isolation and Validation of Cobra Cytotoxins

**Figure 1:**
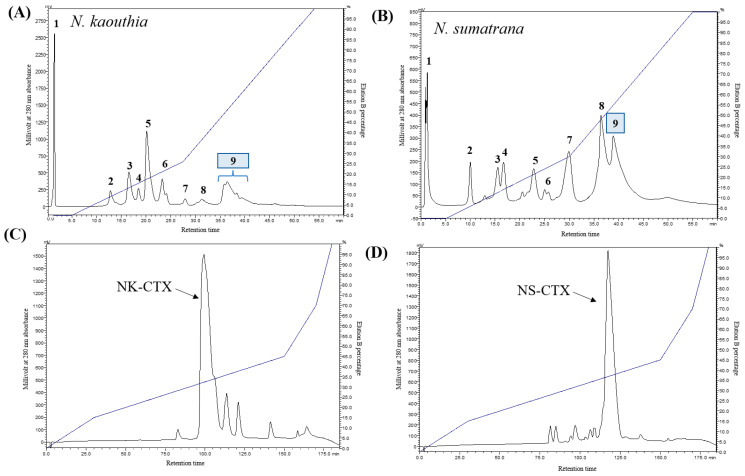
Sequential purification of cytotoxins from *Naja kaouthia* and *Naja sumatrana* venoms via high-performance liquid chromatography (HPLC). Fractionation of *N. kaouthia* (**A**) and *N. sumatrana* (**B**) venoms with Resource S cation-exchange chromatography. Purification of cytotoxin-containing fraction 9 of *N. kaouthia* (**C**) and *N. sumatrana* (**D**) venoms with reverse-phase chromatography. Pointers depict major cytotoxins purified from the cobra venoms.

Cation-exchange HPLC resolved *Naja kaouthia* and *Naja sumatrana* venoms into nine protein fractions (Figure 1A,B). Fraction 9 of both venoms, which contained basic proteins, were further separated by C18 reverse-phase HPLC. The major protein fractions containing CTXs were eluted at similar retention times as previously reported [18], from 90 to 105 min for *N. kaouthia* (NK-CTX; Figure 1C) and from 110 to 125 min for *N. sumatrana* (NS-CTX; Figure 1D).

**Figure 2:**
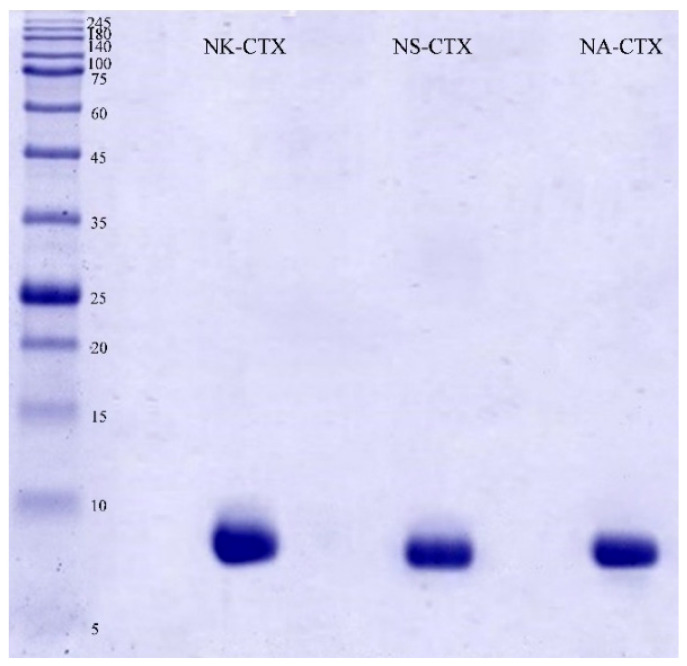
Electrophoretic profile of purified cobra cytotoxins on 15% sodium dodecyl sulfate-polyacrylamide gel electrophoresis (SDS-PAGE) under reducing conditions. Abbreviations: NK-CTX: cytotoxin purified from *N. kaouthia* venom; NS-CTX: cytotoxin purified from *N. sumatrana* venom; NA-CTX: cytotoxin purified from *N. atra* venom.

The purity of the cobra cytotoxins was validated on SDS-PAGE under reducing conditions. The cytotoxins (NK-CTX, NS-CTX, and NA-CTX) each showed a homogeneous band with an estimated molecular weight of approximately 7.0–7.5 kDa (Figure 2).

**Figure 3:**
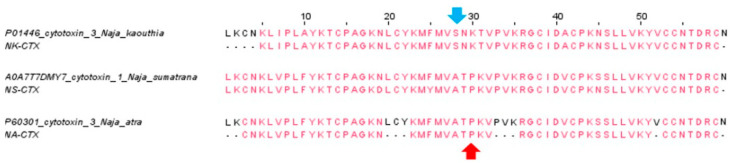
Multiple sequence alignments of cytotoxins identified (NK-CTX, NS-CTX, and NA-CTX), and the sequences of cytotoxin matched in database. Matched amino acid sequences are in red. Dashed lines represent amino acids that were not covered by nano-ESI-LCMS/MS. Arrow in blue depicts Serine28 of S-type cytotoxin, and arrow in red depicts Proline30 of P-type cytotoxin. UniProt ID: P01446 is cytotoxin 3 from *N. kaouthia,* A0A7T7DMY7 is cytotoxin 1 from *N. sumatrana*, P60301 is cytotoxin 3 from *N. atra*.

With nano-LCMS/MS, these cytotoxins were identified and annotated as cytotoxin 3 (P01446, *N. kaouthia*) for NK-CTX, cytotoxin 1 (A0A7T7DMY7, *N. sumatrana*) for NS-CTX, and cytotoxin 3 (Cardiotoxin A3; P60301, *N. atra*) for NA-CTX. All identifications were validated with a high protein score (>200) and peptide sequence coverage (85–93%) (Figure 3, Table 1). Mass spectrometric analysis of the tryptic peptides (protein score, scored peak intensity, peptide sequence, mass-to-charge ratio, and spectral intensity) of NK-CTX, NS-CTX, and NA-CTX is provided in Supplementary File S1.

### 2.2. Cytotoxicity of Cobra Venoms and Purified Cytotoxins

**Figure 4:**
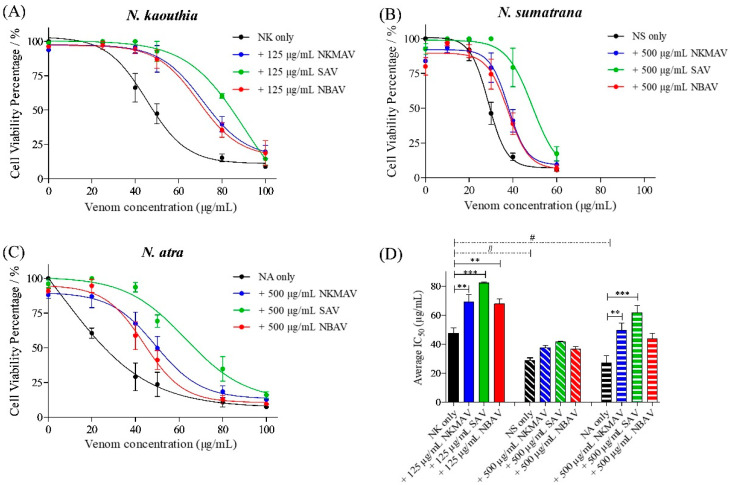
Cytotoxicity of cobra venoms and their neutralization by antivenoms on the CRL-2648 cell lines. Cell viability plot of mouse fibroblast cell line treated with *N. kaouthia* (NK; **A**), *N. sumatrana* (NS; **B**), and *N. atra* (NA; **C**) venoms and their neutralization by cobra antivenoms (NkMAV, SAV, and NBAV). Average IC_50_ of venoms of NK, NS, and NA and neutralization with cobra antivenom (**D**). The assay represents biological and technical triplicates. One-way ANOVA with Tukey’s post hoc test was used in determining statistical significance. ** and *** represents statistical significance between venom IC_50_ and antivenom neutralized IC_50_ within the respective cobra group, *p* < 0.001 and *p* < 0.0001 respectively. # Represents statistical significance between venom IC_50_ of cobra species, *p* < 0.01.

**Figure 5:**
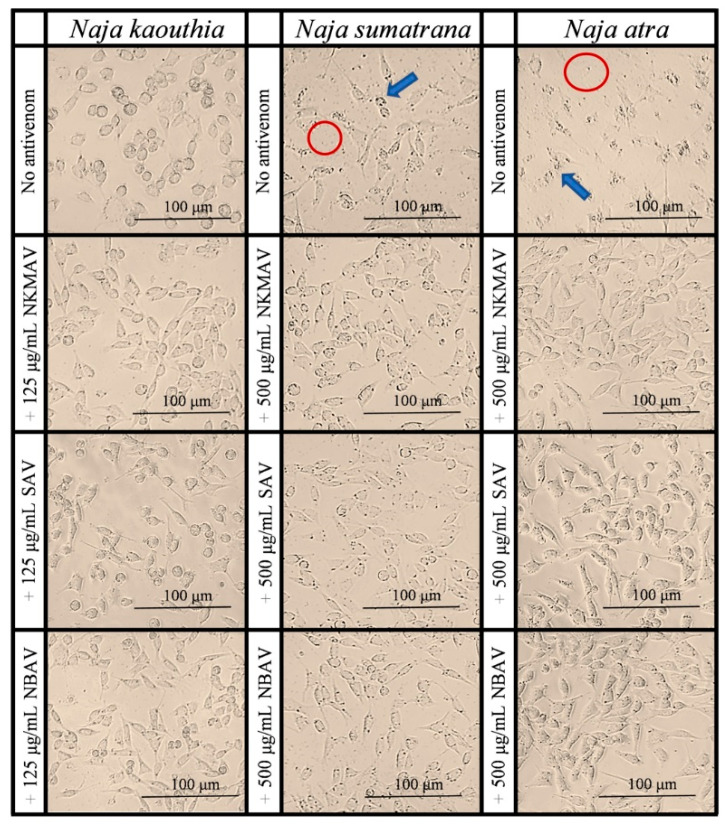
Microscopic observations of CRL-2648 cell lines treated with 20 μg/mL of *N. kaouthia*, *N. sumatrana*, and *N. atra* venoms and its neutralization effect by antivenoms (NkMAV, SAV, or NBAV). Arrow in blue indicates cell vesicles, red ring highlights cell debris.

Figure 4 shows the cytotoxic activities induced by *N. kaouthia*, *N. sumatrana*, and *N. atra* venoms in the mouse subcutaneous fibroblast cells (CRL-2648) and the neutralization effects of the regional antivenoms from Thailand, Vietnam, and Taiwan. Cell viability was impaired by all cobra venoms in a dose-dependent manner. Among the 3 cobra venoms, *N. atra* and *N. sumatrana* venoms were comparably the most cytotoxic (IC_50_ = 26.87 ± 8.86 μg/mL and 28.83 ± 2.86 μg/mL, respectively), while the cytotoxic potency of *N. kaouthia* venom was significantly lower (IC_50_ = 47.40 ± 6.50 μg/mL; *p* < 0.01) (Table 2). The venom-induced cytotoxicity corroborated with microscopic examination, where *N. atra* and *N. sumatrana* venoms caused more prominent alterations in cell morphology than *N. kaouthia* venom after 8 h incubation. Cells treated with *N. atra* and *N. sumatrana* venoms showed a more prominent cell shriveling effect, cell vesicles, and cell debris compared to those treated with *N. kaouthia* venom (Figure 5).

**Figure 6:**
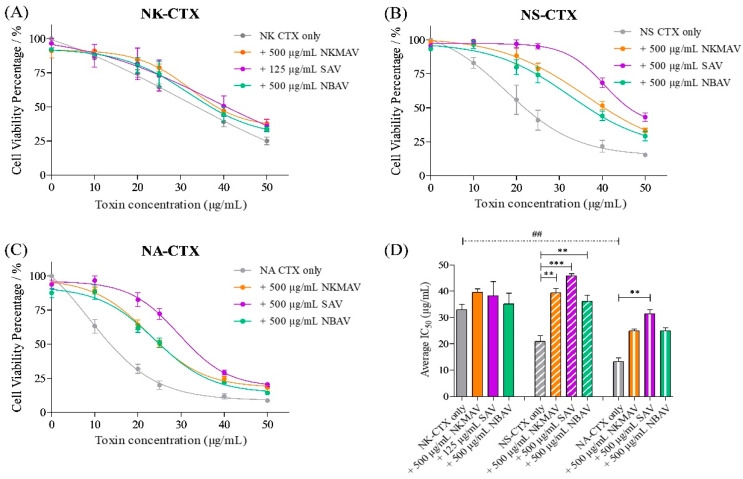
Cytotoxicity of purified cytotoxins from *N. kaouthia* (NK-CTX), *N. sumatrana* (NS-CTX), and *N. atra* (NA-CTX) venoms and neutralization with NkMAV, SAV, and NBAV on CRL-2648 cell line. Cell viability plot of NK-CTX (**A**), NS-CTX (**B**), and NA-CTX (**C**) in mouse fibroblast cell lines and neutralization with NkMAV, SAV, and NBAV. Average IC_50_ of cobra cytotoxins, NK-CTX, NS-CTX, and NA-CTX and neutralization with antivenom (**D**). The assay represents biological and technical triplicates. One-way ANOVA with Tukey’s post hoc test was used in determining statistical significance. ** and *** represents statistical significance between venom IC_50_ and antivenom neutralized IC_50_ within the respective cobra group, *p* < 0.001 and *p* < 0.0001 respectively. ## represents statistical significance between venom IC_50_ of cobra species, *p* < 0.001.

**Figure 7:**
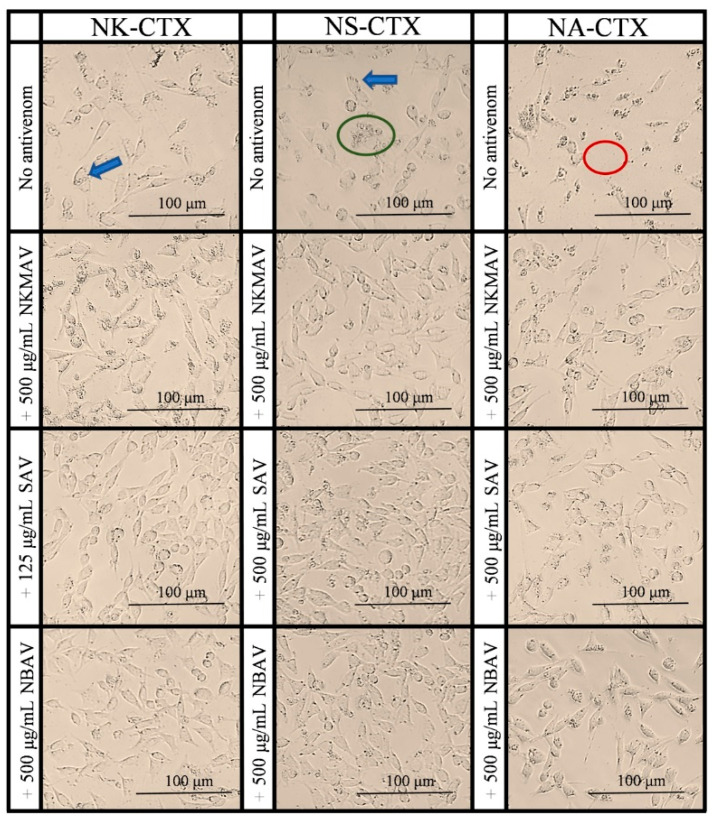
Microscopic observations of CRL-2648 cell lines treated with 20 μg/mL of purified cytotoxins from *N. kaouthia*, *N. sumatrana*, and *N. atra* and its neutralization effect by antivenoms (NkMAV, SAV, or NBAV). Arrow in blue shows cell vesicles, green ring shows cell aggregation, red ring shows cell debris.

Under the same experimental conditions, the cells were treated with the purified cobra cytotoxins (NK-CTX, NS-CTX, and NA-CTX), and the findings revealed a dose-dependent reduction in cell viability (Figure 6). The cytotoxic activities exerted by the purified cytotoxins were significantly higher than their corresponding crude venoms at all doses by 1.5–2.0 folds (*p* < 0.05). NA-CTX was found to be most cytotoxic (IC_50_ = 13.17 ± 2.72 μg/mL), followed by NS-CTX (IC_50_ = 20.90 ± 3.73 μg/mL) and NK-CTX (IC_50_ = 32.90 ± 3.11 μg/mL) (*p* < 0.01). The microscopic morphology of cells was altered to varying degrees by the respective cytotoxins, as shown in Figure 7. Consistent with the varying IC_50_ values, while NK-CTX and NS-CTX treated cells showed increased cell vesicles and cell aggregation, cells treated with the more cytotoxic NA-CTX had complete loss of cell morphology, leaving substantial cell debris (Figure 7).

### 2.3. Neutralization of Cytotoxicity by Antivenoms

The neutralization efficacy of antivenom was determined with an experimental design adapted from Liu et al. [32], quantifying the difference in IC_50_ between the neutralizing group and control (no antivenom) group. Treatments with antivenom effectively reduced the cytotoxicity of cobra venoms and their purified CTXs, as evidenced by the right-shifted cell viability curves and increased IC_50_ values (Figure 4 and Table 2). All 3 antivenoms tested, i.e., NkMAV, SAV, and NBAV were able to reduce the cytotoxicity of *N. kaouthia* venom at varying degrees with 125 µg/mL antivenom concentration (Figure 4A). However, 500 μg/mL antivenom treatments were required to produce noticeable changes in IC_50_ against *N. sumatrana* and *N. atra* venoms (Figure 4B,C). The efficacy levels of different antivenoms against the three venoms were compared in terms of Potency (Pc), which an amount of venom or cytotoxin (in mg) is completely neutralized by a unit amount (g) of antivenom protein, showing at least 1:1000 ratio of venom/CTX to antivenom required for stoichiometric neutralization. All antivenoms exhibited high potencies against *N. kaouthia* venom-induced cytotoxicity, with SAV being the most efficacious (Pc = 278.16 mg/g), followed by NkMAV (Pc = 174.16 mg/g) and NBAV (Pc = 134.40 mg/g) (Table 2). The antivenoms were moderately efficacious against the cytotoxicity of *N. atra* venom (Pc ~33.32–69.86 mg/g, with SAV > NkMAV > NBAV) but weak against the *N. sumatrana* venom (Pc ~15.34–25.34 mg/g, with SAV > NkMAV > NBAV).

In the neutralization study of CTX-induced cytotoxicity, a high dose of antivenom (500 μg/mL) was required to reduce the cytotoxic effects of cytotoxins purified from *N. kaouthia*, *N. sumatrana,* and *N. atra* venoms, except for SAV in which a dose of 125 µg/mL of antivenom protein was adequate to effect neutralization (Table 2). SAV also showed an overall higher potency against the CTXs of individual venoms (Pc of 42.64 mg/g against NK-CTX, 49.74 mg/g against NS-CTX, and 36.66 mg/g against NA-CTX), outperforming the other antivenoms. In comparison between NkMAV and NBAV, both were similarly efficacious against NS-CTX (Pc ~30–37 mg/g) and NA-CTX (Pc ~23 mg/g), while against NK-CTX, NkMAV was much more potent (Pc = 13.20 mg/g) than NBAV (Pc = 4.54 mg/g).

## 3. Discussion

Envenoming caused by cobras (*Naja* spp.) is characterized by two hallmark pathologies: systemic neurotoxicity, which is typically the cause of death, and local cytotoxicity, which often leads to tissue necrosis and permanent disabilities [3,5,36,37]. The severity of neurotoxicity is known to correlate with the amount or abundance of alpha-neurotoxins (post-synaptic blocking neurotoxins) in the cobra venoms [38], but the local tissue effect may not necessarily conform to the amount of cytotoxins/cardiotoxins (CTX), suggesting varying cytotoxic activity of different CTX forms, and/or the presence of other modulatory toxins variably evolved in the different lineages of cobras [39]. The present study showed that the venoms of *N. sumatrana*, *N. kaouthia*, and *N. atra*, three medically important cobra species in Asia known for causing severe local necrosis, exert potent cytotoxicity (with low IC_50_ < 50 μg/mL) when investigated in vitro in the mouse subcutaneous fibroblast cell line (CRL-2648). The degree of cytotoxicity of the three venoms appears to reflect the abundance of CTX in the venoms. The high cytotoxicity of *N. atra* and *N. sumatrana* venoms (IC_50_ = 26–28.83 μg/mL) is consistent with the higher CTX abundance (~50% of total venom proteins) reported in the venom proteomes [12,40] venoms, whereas the *N. kaouthia* venom (IC_50_ ~ 47 μg/mL) had a lower abundance of CTX (~28% of total venom proteins) [41]. Comparatively, the venoms of African spitting cobras, such as *Naja nigricollis, Naja mossambica,* and *Naja pallida*, have relatively higher CTX abundances (64–72%) [42] and thus higher cytotoxicity when examined in the rat myogenic cell line (C2C12) (IC_50_ of ~15 μg/mL) [33]. It has been proposed that the enhanced cobra venom cytotoxicity facilitates the evolution of the defensive spitting behavior of cobras, especially the African spitters from the subgenus of *Afronaja* [39]. The phenotype of venom-spitting behavior and highly cytotoxic venom is in sharp contrast to the African non-spitting cobras (subgenus: *Uraeus*), which cause minimal or negligible local envenoming effect and have lower cytotoxicity [39,43,44], presumably because the CTX of the non-spitting cobras of *Uraeus* subgenus are structurally atypical, containing a substitution of histidine at the 4th amino acid residue [45,46,47], a CTX “variant” reported to exhibit low cytotoxicity [48,49]. The association between venom-spitting behavior and high venom cytotoxicity is, however, weak when examined in the Asiatic cobras (subgenus: *Naja*), as also shown in this study. Notably, the spitting Philippine Cobra, *Naja philippinensis* causes minimal local envenoming effect and has a venom with low cytotoxicity [38,50], while *N. atra* and *N. kaouthia*, conventionally known to be non-spitting, have highly cytotoxic venoms and cause severe tissue necrosis in envenomings much as the typical spitter *N. sumatrana* does [5,51,52]. Nevertheless, it is noteworthy that both *N. atra* and *N. kaouthia* are increasingly perceived as “partial” spitting cobras, where a few specimens have been found to be able to eject venom through the fangs, facilitated with a physical maneuver to enable forward spitting [53,54]. The spitting behavior in *N. atra* and *N. kaouthia* is rare, however, and it lacks the precision of typical spitting cobras.

Our findings of the high cytotoxic activity of *N. kaouthia*, *N. sumatrana*, and *N. atra* venoms are congruent with the clinical phenotype of local envenoming caused by these species (manifested as severe local necrosis), irrespective of their inclination of venom spitting. Further characterization revealed that the purified cytotoxins, i.e., NK-CTX, NS-CTX, and NA-CTX have IC_50_ that is lower than their corresponding venoms, indicating their role as the major cytotoxic components in the venoms. The CTX cytotoxicity is variable among the three cobra species, with the most potent being NA-CTX, followed by NS-CTX and NK-CTX, suggesting structural variation that influences the intrinsic activity of these homologous proteins. The cytotoxicity of CTX also varies depending on the target cells, as NA-CTX exhibited low IC_50_ (IC_50_ = 6.82 μg/mL) in human leukemic myeoloblast cell line (HL60), showing high CTX-specificity toward human cell line compared to murine cell line (IC_50_ = 26.87 μg/mL). The more cytotoxic NA-CTX and NS-CTX belong to the P-type CTX, whereas the relatively less cytotoxic NK-CTX is an S-type CTX. P-type CTXs are generally more cytotoxic than S-type CTXs due to higher lipid-binding activity and deeper penetration into phospholipid bilayers, which readily destabilizes the membrane architecture, causing pore formation and cytolysis [15,17]. This is also supported by the differences in cytotoxicity between NS-CTX (P-type) and NK-CTX (S-type) in a variety of human cell lines of breast (MCF-7), lung (A549), and prostate (PC-3) cancers reported recently [18]. The cytotoxin subtypes (P- and S-type), however, seem independent from the systemic lethality as shown in vivo, where NS-CTX was the most lethal (P-type, LD_50_ = 1.13 μg/g), followed by NK-CTX (S-type, LD_50_ = 1.41 μg/g), and NA-CTX (P-type, LD_50_ = 2.12 μg/g) [32,55,56]. Therefore, the role of CTX subtypes mainly reflects the degree of local tissue cytotoxicity and necrosis evident from the extensive tissue necrosis from *N. sumatrana* and *N. atra* envenomation [32,51].

The findings of distinct CTX with high cytotoxicity in the three cobra venoms underscores the need to evaluate antivenom efficacy in neutralizing not only the whole venoms but also the CTXs specific to the respective venoms. In the neutralization study, the antivenoms NkMAV, SAV, and NBAV demonstrated variable efficacy against the venom- and CTX-induced cytotoxicity, with SAV being the most potent, followed by NkMAV and NBAV. The cross-neutralization capability of the different antivenoms suggests conserved epitopes among the CTXs studied, while their varying efficacy could be due to factors associated with the CTXs and/or the antivenom formulation. The subtypes and abundances of CTX in different cobra venoms are variable, and this can result in diverse immunogenicity and antigenicity of the venom proteins and their neutralization by antivenom [12,57]. The findings showed that between both monovalent antivenoms (NkMAV and SAV), SAV raised against the Vietnamese *N. kaouthia* was more potent (by 1.6 folds) in neutralizing the cytotoxicity induced by *N. kaouthia* venom. This is possibly attributed to the higher abundance of CTX (44.9% of total venom proteins) in the Vietnamese *N. kaouthia* venom compared to the CTX content in the Thai *N. kaouthia* venom (27.6%) [41], which therefore elicited a higher antibody titer in SAV against the CTXs. Furthermore, the content or amount of neutralizing antibodies that specifically target cobra CTX may vary between different antivenom formulations. Naturally, the specific CTX-neutralizing antibody content is anticipated to be higher in a monovalent (mono-specific) antivenom compared with a poly-specific product per mass unit of protein. In comparison, NBAV showed generally lower neutralization potency against the venoms and CTXs tested compared with the monovalent SAV and NkMAV. This is perhaps because NBAV has a proportionally lower antibody titer in neutralizing the cytotoxic components as it is a bivalent product raised against the Taiwanese *N. atra* and *B. multicintus*, the latter of which is an elapid whose venom lacks cytotoxic properties and contains no CTX [32,58]. This is also supported by an earlier study that showed a lower immunocapturing activity of NBAV against the CTX of Taiwanese *N. atra* (<20%) in contrast to SAV, which showed higher immunocapturing activity of >50% [32]. The suggestion of non-monovalent antivenoms having a lower content of specific neutralizing antibodies is also consistent with the observation from another recent study, which showed that the bivalent NBAV is less potent in neutralizing *B. multicinctus* venom compared to a regional *B. multicinctus* monovalent antivenom product [58]. Additionally, the cytotoxin subtypes seem to play a vital role in neutralization as NBAV (raised against the Taiwanese *N. atra*, possessing P-type cytotoxin) exhibited low neutralization potency (Pc = 4.54 mg/g) against the S-type NK-CTX, highlighting the need for cytotoxins subtype considerations in antivenom development.

When compared across the three venoms tested, the three cobra antivenoms consistently showed limited neutralization activity against the cytotoxicity induced by the heterologous *N. sumatrana* venom (Pc = 15.34–25.34 mg/g) despite having modest neutralization efficacy against the NS-CTX (Pc = 30.40–49.74 mg/g). The discrepancy could be due to the presence of toxic neutral PLA_2_ in the *N. sumatrana* venom, which can act synergistically with CTX to potentiate the venom cytotoxicity [59]. The antivenoms tested (raised against *N. kaouthia* and *N. atra)* were probably less effective against this form of toxic PLA_2_ found uniquely in *N. sumatrana* venom, as the PLA_2_s present in *N. kaouthia* and *N. atra* venoms are primarily the non-lethal acidic forms [32,55,56,60]. Moreover, the *N. sumatrana* venom exhibited a much higher PLA_2_ enzymatic activity (enzymatic rate = 82.11 nmol/min/mg) than *N. kaouthia* and *N. atra* venoms (enzymatic rates < 40 nmol/min/mg) [61], a characteristic which may have a synergistic effect on the CTX activity of *N. sumatrana* venom, rendering the reduced efficacy of the antivenoms.

Despite the present findings showing that the three regional antivenoms generally had low efficacy against the in vitro cytotoxicity induced by the cobra venoms and cytotoxins, WHO and Ministry of Health guidelines recommend the use of antivenom on patients exhibiting prominent local cytotoxicity involving more than half of the bitten extremity, rapid swelling extension beyond ankles or wrists within hours, and development of enlarged and tender lymph node draining of the bitten limb [3,62]. Clinical reports of the use of these antivenoms in treating cytotoxicity have been inconsistent, with Vietnamese patients reporting neither mortality nor morbidity with SAV therapy [29], while Taiwanese patients still develop necrosis and require surgical interventions despite early (<6 h) NBAV administration [51]. Nevertheless, antivenom effectiveness is limited in treating tissue necrosis probably due to limited immunogenicity and antigenicity of CTX in view of their small molecular sizes (60–70 amino acid residues) and variable amounts (abundances) in the venoms that reduce the efficiency of eliciting specific antibody production against the toxins during hyperimmunization. In addition, the formulation of an antivenom product as being monovalent or polyvalent probably also has an impact on its neutralization potency against the venom cytotoxicity.

## 4. Conclusions

The present study elucidated the differential cytotoxicity of the three medically important Asiatic cobras (*N. kaouthia*, *N. atra*, and *N. sumatrana*) venoms and their purified cytotoxins applying an in vitro model of mouse fibroblast cell line (CRL-2648) which can be considered prior to the use of the in vivo tissue necrosis assay. The findings underscore the need to improve antivenom efficacy for the neutralization of cytotoxicity induced by cobra CTXs which are small proteins with variable abundances and subtypes across different species. It is suggested that innovative approaches such as the use of toxin-enriched immunogens [63], synthetic or recombinant species-specific antivenoms [64], or small molecule inhibitors [65,66] should be further explored for better treatment of local tissue-damaging effects. Further research should also aim to establish the correlation of cytotoxicity neutralization study between in vitro and in vivo models and how this can be clinically translated for improved treatment outcome of local tissue toxicity in cobra bite envenomings.

## 5. Materials and Methods

### 5.1. Venoms and Antivenoms

*Naja kaouthia* venom was supplied by the Queen Saovabha Memorial Institute (QSMI), Bangkok, Thailand. *Naja sumatrana* venom was a sample collected from adult snakes in Seremban, Peninsular Malaysia. *Naja atra* venom was obtained from a local snake farm in Tainan, Taiwan. All venoms were pooled samples of more than 10 adult snakes, lyophilized, and stored at −20 ℃ until use. *N. kaouthia* and *N. sumatrana* venoms were subjected to the purification of cytotoxins as described below. The cytotoxin of *N. atra* was provided by the National Health Research Institutes, Taiwan, obtained through a purification process as reported earlier by Liu et al. [32].

Thai *Naja kaouthia* Monovalent Antivenom (NkMAV), Vietnamese *Naja kaouthia* Monovalent Antivenom (SAV, as Snake Antivenom per manufacturer’s labeling), and Taiwanese Neuro Bivalent Antivenom (NBAV) were used in the present study. NkMAV (batch no.: NK00116) is a monospecific antivenom raised against Thai *Naja kaouthia* venom, produced by the Queen Saovabha Memorial Institute (QSMI), Bangkok, Thailand. SAV (batch no.: 039-00-18) is a monospecific antivenom raised against Vietnamese *Naja kaouthia* venom, produced by the Venom Research Unit, Ho Chi Minh City, Vietnam. NBAV (batch no.: 61-06-0002) is a bivalent antivenom raised against Taiwanese *Bungarus multicintus* and *Naja atra* venoms, manufactured by Taiwan Central for Disease Control, Taipei, Taiwan. All antivenoms were lyophilized F(ab’)_2_ derived from horse antisera. NkMAV and NBAV are lyophilized types of antivenoms that were reconstituted in 10 mL normal saline per vial prior to use.

### 5.2. Resource S Cation-Exchange and C_18_ Reverse-Phase High-Performance Liquid Chromatography

Cytotoxins of *N. kaouthia* (NK-CTX) and *N. sumatrana* (NS-CTX) venoms were purified with sequential fractionation using Resource S cation-exchange and C_18_ reverse-phase high-performance liquid chromatography using Shimadzu LC-20AD High-Performance Liquid Chromatography (HPLC) system (Kyoto, Japan), as previously described [18,56]. Briefly, the venom samples were reconstituted in 20 mM 2-(N-morpholino)ethanesulfonic acid (MES; eluent A; pH 6) and subjected to Resource S cation-exchange (GE Healthcare, Sweden) chromatographic fractionation. The HPLC system was pre-equilibrated with eluent A, and elution was carried out with 0.8 M sodium chloride in 20 mM MES (eluent B; pH 6) at a linear gradient of 0 to 30% eluent B from 5 to 40 min, followed by 30 to 100% from 40 to 65 min. The flow rate was kept at 1 mL/min, and the elution was monitored at 280 nm absorbance. The protein fractions were manually collected and subjected to buffer exchange using Vivaspin 20 (5000 MWCO) polyethersulfone membrane (Sartorius, Göttingen, Germany).

The retentates from cation-exchange chromatography, which contains NK-CTX and NS-CTX, were subjected to reverse-phase fractionation via LiChrospher WP 300 C_18_ (5 µm pore size) reverse-phase column (Kyoto, Japan). In brief, the retentates were reconstituted in 0.1% trifluoroacetic acid (TFA) in ultrapure water (solvent A). Column pre-equilibration was performed with solvent A, and retentates were subsequently separated with 0.1% TFA in acetonitrile (solvent B), applying linear gradient elution as follows: 5% for 10 min, 5 to 15% for 20 min, 15 to 45% for 120 min, and 45 to 70% for 20 min. The flow rate was maintained at 1 mL/min, and the elution was monitored at 215 nm absorbance. Protein fractions were collected manually, freeze-dried, and stored at −20 ℃ until further use.

### 5.3. Sodium Dodecyl Sulfate-Polyacrylamide Gel Electrophoresis (SDS-PAGE)

The cytotoxins of *N. kaouthia* (NK-CTX), *N. sumatrana* (NS-CTX), and *N. atra* (NA-CTX) were subjected to 15% SDS-PAGE under reducing conditions at 90 V for 2.5 h. ExcelBand™ 3-colour Broad Range Protein Marker (Smobio, Hsinchu, Taiwan) was used for molecular weight estimation. Proteins were visualized by staining the gel with Coomassie Brilliant Blue R-250 (Sigma-Aldrich, St. Louis, MO, USA). The molecular weights of the cytotoxins were estimated using GelAnalyzer (Version 19.1) [67].

### 5.4. Nano-Electrospray Ionization Liquid Chromatography-Tandem Mass Spectrometry (Nano-ESI LC-MS/MS)

A total of 5 micrograms of the purified NK-CTX, NA-CTX, and NS-CTX were reduced by dithiothreitol (DTT), alkylated with iodoacetamide (IA), and subjected to in-solution digestion with 0.1 μg/μL of MS grade Pierce trypsin protease (trypsin to sample ratio 1:10; Thermo Scientific Pierce, Rockford, IL, USA). Peptide desalting and extraction were carried out with Millipore ZipTip^®®^ C_18_ pipette tip (Merck, Branchburg, NJ, USA). The extracted peptides then underwent nano-electrospray ionization-liquid chromatography-tandem mass spectrometry (nano-ESI-LC-MS/MS) with Agilent 1200 HPLC-Chip/MS Interface, paired with Agilent 6520 Accurate-Mass Q-TOF LC/MS system (Santa Clara, CA, USA). A total of 0.1% formic acid in water and 90% acetonitrile in water with 0.1% formic acid were utilized, respectively, as eluting solvents A and B. Samples were then analyzed with Agilent HPLC-Chip II: G42040-62010, comprised of 160 nL enrichment column and a 75 μm × 150 mm analytical column packed with Agilent ZORBAX 300SB-C18, 5 µm, 300Å. Sample elution was carried out with the same linear gradient as previously described [68]: 3–50% solvent B for 30 min, 50–95% solvent B for 2 min, and 95% solvent B for 5 min. The drying gas flow rate was set to 5 l/min at 325 ℃, and the ion polarity was set to positive. Fragmentor voltage was adjusted to 175 V while capillary voltage was 1995 V. Spectra acquisition was assigned at MS 110–3000 *m/z* and 50–3000 *m*/*z* scan range. The precursor was adjusted to include a doubly or more charged state, apart from reference precursor ions (922.0098 *m/z* (z = 1) and 121.0509 (z = 1). MH^+^ range between 600 and 4000 Da was acquired and processed with Agilent Spectrum Mill MS Proteomics Workbench software packages (B.04.00). Peptide sequences obtained were searched against the non-redundant NCBI database (taxonomy: Serpentes, taxid: 8570) (https://www.ncbi.nlm.nih.gov/Taxonomy/Browser/wwwtax.cgi?mode=Info&id=8570, accessed on 22 July 2020). Carbamidomethylation was set as a fixed modification. The identified proteins were validated with the following parameters: peptide score filter > 6, protein score filter > 10, and scored peak intensity (SPI) > 60%.

### 5.5. Multiple Sequence Alignment

Tryptic peptides were aligned with annotated toxins with the highest homology. The annotated toxin sequences were obtained from the UniProtKB depository. Multiple sequence alignment was conducted with Jalview [69] and MUSCLE [70] software (version 2.10.5).

### 5.6. Cell Culture

The murine subcutaneous fibroblast cell line (L cells; ATCC^®®^ CRL-2648) was supplied by American Type Culture Collection (ATCC; Manassas, VA, USA). The cells were cultured in Dulbecco’s Modified Eagle’s Medium (DMEM; Nacalai tesque, Kyoto, Japan) with 10% fetal bovine serum (TICO Europe, Amstelveen, The Netherlands) and 1% penicillin-streptomycin (Nacalai tesque; Kyoto, Japan) in a humidified atmosphere of 5% CO_2_ and 37 ℃ in T25 flasks. The cells were passaged in accordance with ATCC guidelines using trypsin-EDTA (0.05% trypsin and 0.02% EDTA; Nacalai tesque, Kyoto, Japan).

### 5.7. In Vitro Cytotoxicity Induced by Cobra Venoms and CTX

In vitro assay protocols were modified protocols adapted from Liu et al. [32] and Chong et al. [18]. The cytotoxic activities of cobra venoms and their purified cytotoxins were tested with 3-(4,5-dimethylthiazol-2-yl)-2,5-diphenyltetrazolium bromide (MTT; Sigma-Aldrich, MO, USA). A hundred microliters of the aliquot of cells (15,000 cells/well) were seeded into 96-well microtiter plates 24 h prior to venom or cytotoxin treatment. The cells were treated with 200 μL of serially diluted (10 to 100 μg/mL) venom or purified cytotoxin (NK-CTX, NS-CTX, NA-CTX) of the respective cobra species in a complete medium. Cells without venom served as the negative control. The cells were then treated for 8 h, in conjunction with a previous report by Omran et al. [71] demonstrating the highest cytotoxicity in *N. haje* venom at 8 h treatment. After incubation, cytotoxicity was determined by introducing 10% MTT solution and subsequently incubated in the dark and at 37 ℃ for 3 h. Purple formazans created were then dissolved with 200 μL DMSO, and their absorbance was quantified at 570 nm using Chameleon™ vs. Multilabel microplate reader (Hidex, Turku, Finland). All assays were performed with biological and technical triplicates. The cell viability percentage was calculated according to the formula below:

$$\mathrm{Cell}\;\mathrm{viability}\;\mathrm{percentage}=\frac{\left(\mathrm{Absorbance}\;\mathrm{of}\;\mathrm{experimental}\;\mathrm{sample})\;-\;(\mathrm{Absorbance}\;\mathrm{of}\;\mathrm{blank}\;\mathrm{sample}\right)}{\left(\mathrm{Absorbance}\;\mathrm{of}\;\mathrm{the}\;\mathrm{untreated}\;\mathrm{sample}\right)\;-\;(\mathrm{Absorbance}\;\mathrm{of}\;\mathrm{blank}\;\mathrm{sample})}\times 100\%$$

Half maximal inhibitory concentrations (IC_50_) were then determined for the respective venoms and cytotoxins with GraphPad Prism (Version 5.03) with nonlinear regression analysis, applying the 4-parameter logistic equation for dose-response inhibition study, and the data (encompassing 3 biological and 3 technical triplicates) were expressed as means ± standard error of triplicates.

### 5.8. Neutralization Assay of Cytotoxicity

The cobra venoms or toxins were prepared at a serial dose of 10 to 100 μg/mL as described above for the determination of cytotoxicity. The venom or toxins were pre-incubated with a fixed dose of antivenom treatment (125 μg/mL of SAV, or 500 μg/mL of NBAV and NKMAV) in complete medium at 37 ℃ for 30 min. Subsequently, the mixture was added to the cell-seeded wells. Cytotoxicity and IC_50_ values were determined after 8 h with the MTT assay as described above, also performed in biological and technical triplicates.

The in vitro efficacy of cytotoxicity neutralization by antivenom, termed Potency (Pc), was interpreted in terms of the absolute change in IC_50_ of the venom or toxin affected by the antivenom treatment given at a normalized dose. This was calculated with the following formula modified from a previous study [32]:

$$\mathrm{Potency}\;\left(\mathrm{Pc}\right)\left(\mathrm{mg}/g\right)=\frac{\left[{\mathrm{IC}}_{50}\left(\mathrm{Treated}\right)-{\mathrm{IC}}_{50}\left(\mathrm{Untreated}\right)\right]\;\mathrm{in}\;\mu g/\mathrm{mL}\;}{\left[\mathrm{Antivenom}\;\mathrm{concentration}\right]\;\mathrm{in}\;\mathrm{mg}/\mathrm{mL}}$$

The IC_50_ values of the venom/cytotoxin-induced cytotoxicity tests with and without antivenom treatment. The calculated difference in IC_50_ was then normalized by the dose of antivenom (in mg/mL) used in the corresponding treatment group. The resultant Pc value represents the amount of venom or cytotoxin (in mg) neutralized per gram of antivenom protein under the experimental conditions to effect the change in IC_50_ between treated and untreated groups.

### 5.9. Statistical Analysis

One-way ANOVA with Tukey’s multiple *post hoc* comparison test on GraphPad Prism (Version 5.03) was used in comparing cell viability. Differences in means were statistically significant when *p*-value < 0.05.

## Figures and Tables

**Table 1:** Protein identification of cobra cytotoxins from *Naja kaouthia* (NK-CTX), *Naja sumatrana* (NS-CTX), and *Naja atra* (NA-CTX) by nano-ESI-LCMS/MS.

Protein Designated Identity	Protein Score	Protein Name Matched	Accession Code (Species)	Matched Distinct Peptide Sequences	Coverage (%)
NK-CTX	201.05	Cytotoxin 3	P01446 (*N. kaouthia*)	LIPLAYK LIPLAYKTCPAGK LVPLFYK MFMVAAPK MFMVATPK MFMVSNK MFMVSNKTVPVK NSLLVKYVCCNTDR SSLLVK TCPAGKNLCYK YVCCNTDR GCIDACPK GCIDACPKNSLLVK	92.7
NS-CTX	252.30	Cytotoxin 1	A0A7T7DMY7 (*N. sumatrana*)	LKCNKLVPLFYK CNKLVPLFYK DLCYK LIPLAYK LVPLFYK LVPLFYKTCPAGK MYMVATPK MYMVATPKVPVK NSLLVK RGCIDVCPK SSLLVK SSLLVKYVCCNTDR YVCCNTDR GCIDVCPK GCIDVCPKSSLLVK	93.3
NA-CTX	208.83	Cytotoxin 3	P60301 (*N. atra*)	CNKLVPLFYK LVPLFYK LVPLFYKTCPAGK MFMVAAPK MFMVATPK MFMVSNK MYMVATPK NSLLVK RGCIDVCPK SSLLVK SSLLVKYVCCNTDR YVCCNTDR GCIDVCPK	85.0

**Table 2:** Cytotoxicity of *N. kaouthia*, *N. sumatrana*, and *N. atra* venoms and NK-CTX, NS-CTX, and NA-CTX in CRL-2648 cells and neutralization by NBAV, NkMAV, and SAV.

Venom/Toxin	IC_50_ (Untreated) ^a^ (μg/mL)	NkMAV			SAV			NBAV		
		IC_50_ (Treated) ^b^ (μg/mL)	Antivenom Concentration (μg/mL)	Pc ^c^ (mg/g)	IC_50_ (Treated) ^b^ (μg/mL)	Antivenom Concentration (μg/mL)	Pc ^c^ (mg/g)	IC_50_ (Treated) ^b^ (μg/mL)	Antivenom Concentration (μg/mL)	Pc ^c^ (mg/g)
*N. kaouthia* (Thailand)	47.40 ± 6.50	69.17 ± 8.39	125	174.16	82.17 ± 0.85	125	278.16	64.20 ± 7.00	125	134.40
*N. sumatrana* (Malaysia)	28.83 ± 2.86	37.47 ± 2.77	500	17.28	41.50 ± 1.25	500	25.34	36.50 ± 2.95	500	15.34
*N. atra* (Taiwan)	26.87 ± 8.86	49.60 ± 8.47	500	45.46	61.80 ± 6.73	500	69.86	43.53 ± 6.48	500	33.32
NK-CTX	32.90 ± 3.11	39.50 ± 2.40	500	13.20	38.23 ± 1.48	125	42.64	35.17 ± 7.10	500	4.54
NS-CTX	20.90 ± 3.73	39.33 ± 2.82	500	36.86	45.77 ± 9.40	500	49.74	36.10 ± 4.08	500	30.40
NA-CTX	13.17 ± 2.72	24.87 ± 1.36	500	23.40	31.50 ± 2.71	500	36.66	24.97 ± 1.95	500	23.60

NkMAV: Thai *Naja kaouthia* monovalent antivenom; SAV: Vietnamese *Naja kaouthia* monovalent antivenom; NBAV: Taiwanese neurobivalent antivenom; IC_50_: Half maximal inhibitory concentration; Pc: Potency. ^a^ IC_50_(Untreated), defined as half maximal inhibitory concentration of venom/cytotoxin without antivenom. ^b^ IC_50_(Treated), defined as half maximal inhibitory concentration of venom/cytotoxin with antivenom treatment. ^c^ Pc, Potency, defined as amount of venom/cytotoxin, in mg, neutralized by 1 g of antivenom.

## Data Availability

The data presented in this study are available in this article and Supplementary Materials.

## Supplementary Materials

The following supporting information can be downloaded at: https://www.mdpi.com/article/10.3390/toxins14050334/s1, Supplementary File S1: nano-LC-MS/MS of purified cytotoxins from *Naja kaouthia* (NK-CTX), *Naja sumatrana* (NS-CTX), and *Naja atra* (NA-CTX)
